# Molecular and Clinical Features of Hospital Admissions in Patients with Thoracic Malignancies on Immune Checkpoint Inhibitors

**DOI:** 10.3390/cancers13112653

**Published:** 2021-05-28

**Authors:** Dan Zhao, Haiqing Li, Isa Mambetsariev, Chen Chen, Rebecca Pharaon, Jeremy Fricke, Angel R. Baroz, Prakash Kulkarni, Yan Xing, Erminia Massarelli, Marianna Koczywas, Karen L. Reckamp, Kim Margolin, Ravi Salgia

**Affiliations:** 1 Department of Medical Oncology and Therapeutics Research, City of Hope National Medical Center, Duarte, CA 91010-3000, USA; DZhao3@mdanderson.org (D.Z.); Imambetsariev@coh.org (I.M.); rpharaon@coh.org (R.P.); jfricke@coh.org (J.F.); abaroz@coh.org (A.R.B.); pkulkarni@coh.org (P.K.); yxing@coh.org (Y.X.); emassarelli@coh.org (E.M.); MKoczywas@coh.org (M.K.); Karen.Reckamp@cshs.org (K.L.R.); kmargolin@coh.org (K.M.); 2 Integrative Genomics Core, Beckman Research Institute, City of Hope Medical Center, Duarte, CA 91010-3000, USA; hali@coh.org; 3 Department of Computational & Quantitative Medicine, Beckman Research Institute, City of Hope National Medical Center, Duarte, CA 91010-3000, USA; 4 Applied AI and Data Science, City of Hope National Medical Center, Duarte, CA 91010-3000, USA; chechen@coh.org; 5 Department of Medicine, Cedars-Sinai Medical Center, Los Angeles, CA 91010-3000, USA

**Keywords:** lung cancer, checkpoint inhibitors, immune-related adverse events (irAEs), admissions, genomic alterations, next-generation sequencing, overall survival

## Abstract

**Simple Summary:**

Lung cancer immunotherapy has many complications and hospitalizations that often occur in non-small cell lung cancer (NSCLC) while on immunotherapy due to adverse events or other factors. The molecular and clinical profiles of these patients are often not well-defined, and the aim of our retrospective study is to better understand these clinical and molecular features. We evaluated a cohort of 90 stage IV thoracic malignancy patients who had hospital admissions after treatment with immune checkpoint inhibitors. We identified a relationship between immune-related adverse events (irAEs) and molecular markers that showed unique survival outcomes, as well as a significant overall survival improvement in patients who required discontinuation or interruption of immunotherapy due to irAEs.

**Abstract:**

Lung cancer patients undergoing systemic treatment with immune checkpoint inhibitors (ICIs) can lead to severe immune-related adverse events (irAEs) that may warrant immediate hospitalization. Patients with thoracic malignancies hospitalized at City of Hope while undergoing treatment with ICIs were identified. Pathology and available next-generation sequencing (NGS) data, including the programmed death-ligand 1 (PD-L1) status and clinical information, including hospitalizations, invasive procedures, and the occurrence of irAEs, were collected. Unpaired T-tests, Chi-square/Fisher’s exact test, and logistic regression were used to analyze our cohort. The overall survival (OS) was calculated and compared using univariate and multivariate COX models. Ninety patients with stage IV lung cancer were admitted after ICI treatment. Of those patients, 28 (31.1%) had documented irAEs. Genomic analyses showed an enrichment of *LRP1B* mutations (*n* = 5/6 vs. *n* = 7/26, 83.3% vs. 26.9%; odds ratio (OR) (95% confidence interval (CI): 13.5 (1.7–166.1); *p* < 0.05) and *MLL3* mutations (*n* = 4/6, 66.7% vs. *n* = 5/26, 19.2%; OR (95% CI): 8.4 (1.3–49.3), *p* < 0.05) in patients with irAE occurrences. Patients with somatic genomic alterations (GAs) in *MET* (median OS of 2.7 vs. 7.2 months; HR (95% CI): 3.1 (0.57–17.1); *p* < 0.05) or *FANCA* (median OS of 3.0 vs. 12.4 months; HR (95% CI): 3.1 (0.70–13.8); *p* < 0.05) demonstrated a significantly shorter OS. Patients with irAEs showed a trend toward improved OS (median OS 16.4 vs. 6.8 months, *p* = 0.19) compared to hospitalized patients without documented irAEs. Lung cancer patients who required treatment discontinuance or interruption due to irAEs (*n* = 19) had significantly longer OS (median OS 18.5 vs. 6.2 months; HR (95% CI): 0.47 (0.28–0.79); *p* < 0.05). Our results showed a significant survival benefit in lung cancer patients hospitalized due to irAEs that necessitated a treatment interruption. Patients with positive somatic GAs in *MET* and *FANCA* were associated with significantly worse OS compared to patients with negative GAs.

## 1. Introduction

Immune checkpoint inhibitors (ICIs) targeting programmed cell death protein 1 (PD-1), programmed death-ligand 1 (PD-L1), and cytotoxic T-lymphocyte–associated antigen 4 (CTLA-4) have transformed the landscape of lung cancer treatment. Pembrolizumab, an anti-PD-1 monoclonal antibody, was approved by the US Food and Drug Administration (FDA) as a monotherapy first-line treatment of metastatic non-small cell lung cancer (NSCLC) with PD-L1 expression ≥ 1, and in combination with chemotherapy regardless of PD-L1 status [1,2,3,4]. 

Atezolizumab and durvalumab, anti-PD-L1 monoclonal antibodies, were approved as a first-line treatment in combination with chemotherapy for small cell lung cancer (SCLC) [5,6,7]. Nivolumab, an anti-PD-1 monoclonal antibody, and ipilimumab, an anti-CTLA-4 monoclonal antibody, with or without chemotherapy, were also recently approved for first-line treatment of metastatic lung cancer [8,9,10,11,12]. Durvalumab was approved for consolidation therapy after chemoradiation in unresectable stage III NSCLC, leading to further investigation of ICIs in the neoadjuvant setting [13,14]. 

However, hospital admission during treatment is common in cancer patients undergoing systemic treatment. Distinct toxicity profiles and immune-related adverse events (irAEs) due to ICIs have been widely reported, including skin reactions, thyroid disorders, pneumonitis, colitis, hepatitis, hypophysitis, and myocarditis [15,16]. Other severe adverse events not related to ICIs can emerge as well during treatment and lead to hospitalization. In a meta-analysis of 35 clinical trials involving ICIs, irAEs of grade 3 and above were reported in 14% of patients treated with monotherapy ICIs, 34% with anti-CTLA-4 antibodies, 46% with combination ICI-chemotherapy, and 55% with ICIs combinations [17]. 

Fatality rates were observed in 0.36% of patients treated with PD-1 inhibitors, 0.38% with PD-L1 inhibitors, 1.08% with CTLA-4 inhibitors, and 1.23% with combination therapy of PD-1/PD-L1 and CTLA-4 inhibitors [18]. Patients who expired during PD-1/PD-L1 inhibitor treatment had severe complications, including pneumonitis (35%), hepatitis (22%), and neurotoxicities (15%); a majority of the deaths observed in the CTLA-4 treatment group were due to severe colitis (70%) [18]. 

Several prominent oncologic societies, such as the National Comprehensive Cancer Network (NCCN), American Society of Clinical Oncology (ASCO), and Society for Immunotherapy of Cancer (SITC), have published guidelines on the management of irAEs in the standard clinical setting. However, irAE management in patients who require hospitalization and are steroids-refractory remains problematic [19,20,21]. The characterization of clinical features regarding irAEs and non-irAEs in hospitalized patients may facilitate the understanding and management of toxicities in this setting. 

Previous studies have described associations between several tumor genomic features and the tumor response to ICIs. Notably, a poor tumor response was reported in patients on ICIs with molecular alterations in EGFR or MET [22,23,24]. In addition to a poor response, the development of severe irAEs (especially within 3 months) has been described in a retrospective analysis of EGFR mutated NSCLC patients (15%; 6/41) treated with ICIs followed by osimertinib, although the underlying mechanisms are still poorly understood [25]. However, the development of irAEs has not been established as a predictive marker in measuring responsiveness to ICIs. 

The clinical characterization of irAEs and full assessment of genomic data is necessary to optimize the patient selection criteria for ICI treatment, understand the underlying mechanisms of irAE development, and develop novel strategies to avoid irAEs while maintaining the anti-tumor efficacy [26]. In our retrospective analysis, we collected clinical and molecular information on 90 patients diagnosed with thoracic malignancies who received ICI treatment and were subsequently hospitalized in order to characterize irAE and non-irAE development, evaluate the management of irAEs, and analyze the survival outcomes. 

## 2. Materials and Methods

### 2.1. Patients

Patients with metastatic thoracic malignancies who were hospitalized after receiving ICI treatment (pembrolizumab, nivolumab, atezolizumab, and ipilimumab/nivolumab) in different treatment settings, including standard of care, compassionate use, and clinical trials at City of Hope were reviewed. Ninety patients with histologies, including SCLC, NSCLC, and other thoracic malignancies were identified. Demographic, clinical, and pathological information was collected with approval by the City of Hope institutional review board (IRB #18529). The overall survival (OS) was measured from the start of the ICI treatment to the date of death and calculated, if available, at the study time point. The data cutoff date was 8 November 2018.

### 2.2. Clinical and Molecular Information Collection

Tumor genomic alterations (GAs) were extracted from the available clinical data on next-generation sequencing (NGS) via several platforms, including FoundationOne (Foundation Medicine, Cambridge, MA, USA), Caris (Caris Life Sciences, Phoenix, AZ, USA), Paradigm (Paradigm Diagnostics, Phoenix, AZ, USA), Guardant360 (Guardant, Redwood City, CA, USA), NeoGenomics (NeoGenomics Laboratories, Fort Myers, FL, USA), and City of Hope gene sequencing panels. The PD-L1 (22C3) expression by immunohistochemistry was reported as the tumor proportion score (TPS), which is defined as the percentage of viable tumor cells showing partial or complete membrane staining of ≥1% relative to all viable tumor cells present in the sample. 

Negative PD-L1 expression was defined as <1% of viable tumor cells showing membranous staining. The tumor mutational burden (TMB) was reported and categorized as low (≤5 Muts/Mb), intermediate (6–19 Muts/Mb), or high (≥20 Muts/Mb) by Foundation Medicine. Somatic GAs were sorted by the detected positive rate of GAs among all tested patients (the number of tested patients for each gene varied due to different gene panels in the testing platforms). IrAEs were defined as treatment-related toxicities documented by the admitting physician or primary oncologist and independently confirmed by another physician who reviewed the patient medical charts, including the laboratory, imaging, and pathological evidence. 

The severity of irAEs was documented from grade 1 to 5 as per the National Institute of Health Common Terminology Criteria for Adverse Events (CTCAE), version 4.03. Clinical information, such as lines of therapy; the length of stay (LOS) in hospital; the status of metastatic disease in the brain; the therapy regimen of ICIs; the management of irAEs, including invasive, diagnostic, and therapeutic procedures; and any interruption or discontinuation of ICIs due to irAEs was also collected.

### 2.3. Statistical Analysis 

First, the association of clinical and molecular features with the OS was analyzed using the univariate Cox proportional hazards model. Based on the results of the univariate analysis, clinically and biologically relevant features with statistical significance (cutoff *p*-value of 0.05 with the number of patients, *n* ≥ 5) were selected for the multivariate Cox proportional hazards model. TMB was categorized by Foundation Medicine molecular testing reports. PD-L1 expression was categorized as negative (<1%), and positive (grouped as 1–49% and ≥50%). 

We used the Kaplan–Meier method and log-rank test to estimate the OS, and we compared the survival curves, respectively. The chi-square test, Fisher’s exact test, and logistic regression were used for comparison between patient groups (i.e., patients who had irAEs vs. patients who did not have irAEs). Statistical analyses and data visualization were performed using GraphPad Prism 8 (GraphPad Software, Version 8, Graphpad Holdings, LLC, San Diego, CA, USA) and R (version 3.6.2, R Foundation for Statistical Computing, Vienna, Austria) [27]. All tests were two-sided, and *p* < 0.05 was considered statistically significant. 

## 3. Results

### 3.1. Patients Characteristics 

Ninety patients with stage IV thoracic malignancies underwent admission to the City of Hope after ICI treatment. The dates of ICI treatment initiation were between 6 May 2015, and 6 August 2018. Of those admitted, 28 (31.1%) had documented irAEs, and 62 (68.9%) did not experience any irAEs (Table 1). The most common irAE was pneumonitis (*n* = 10, 11.1%) followed by adrenal insufficiency (*n* = 4, 4.4%), hypothyroidism (*n* = 4, 4.4%), colitis (*n* = 4, 4.4%), liver injury (*n* = 3, 3.3%), nephritis (*n* = 2, 2.2%), infection (*n* = 2, 2.2%), rash (*n* = 2, 2.2%), heart failure (*n* = 1, 1.1%), pancreatitis (*n* = 1, 1.1%), diabetic ketone acidosis (*n* = 1, 1.1%), and arthralgia (*n* = 1, 1.1%). Seven patients (7.8%) experienced multiple irAEs. 

The baseline characteristics of the 90 patients are summarized in Table 2. Disease histologies included 63 patients (70%) with adenocarcinoma, 14 (15.6%) with squamous cell lung cancer, 5 (5.6%) with SCLC, and 8 (8.9%) with other types (1 poorly differentiated tumor including NSCLC, not otherwise specified (NSCLC-NOS), 1 large cell lung cancer, 1 lung atypical carcinoid, 1 adenosquamous tumor, 1 mixed large cell with neuroendocrine tumor, 1 small cell transformed lung adenocarcinoma, 1 mixed adenocarcinoma with large cell neuroendocrine tumor, and 1 mesothelioma). Thirty-five patients (38.9%) had documented brain metastases. The median age was 68.5 years (range 36–88), with 70.5 years in the irAEs group and 67.5 years in the non-irAEs group. 

**Figure 1:**
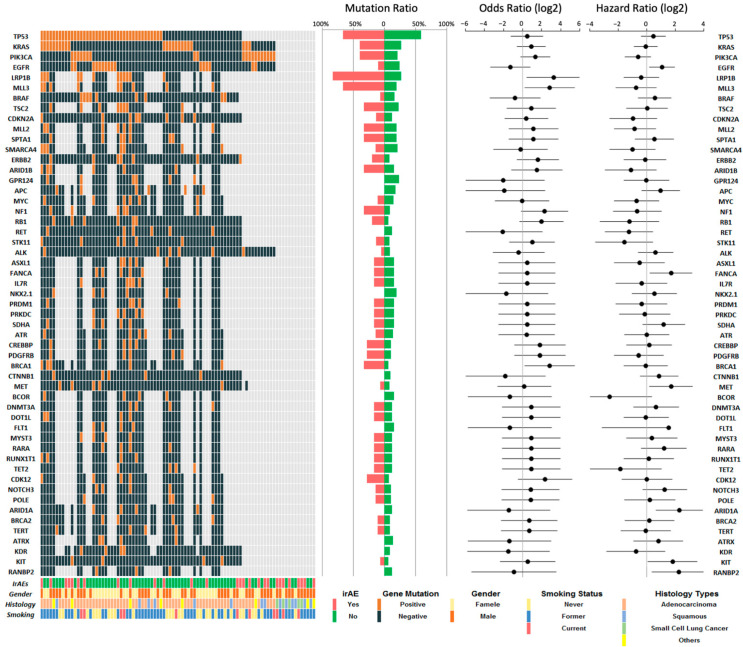
The top detected genomic alterations with OS and irAEs. Oncoplot demonstrating the patient demographic information and top detected genomic alterations in 90 lung cancer patients who were hospitalized during ICI treatment. Mutation rates are shown by patients with irAEs vs. no irAEs. The odds ratios were calculated using univariate logistic regression and hazard ratios with the univariate Cox model. Data visualization and statistical analysis were performed with R.

Gender was similarly divided in our patient population (41 women, 45.6%, and 49 men, 54.4%). Seventy-eight patients (86.7%) received ICIs as monotherapy and 12 (13.3%) received ICIs combined with chemotherapy. The median lines of therapy were two (range one to seven lines). The median LOS was 7 days (range 1–37 days). The smoking history of our cohort confirmed 29 never smokers (32.2%), 50 former smokers (55.6%), and 11 current smokers (12.2%). PD-L1 by IHC was reported in 45 patients: 16 (35.6%) were negative (TPS of <1%), 8 (17.8%) were positive (TPS of 1–49%), and 21 (46.7%) were highly positive (TPS of ≥50%). Seventy-seven patients underwent *EGFR* molecular testing, and 20.8% (*n* = 16) were *EGFR* positive. The mutational landscape of our patient population is shown in Figure 1. 

### 3.2. Clinical Features in irAEs and Non-irAE Population

**Figure 2:**
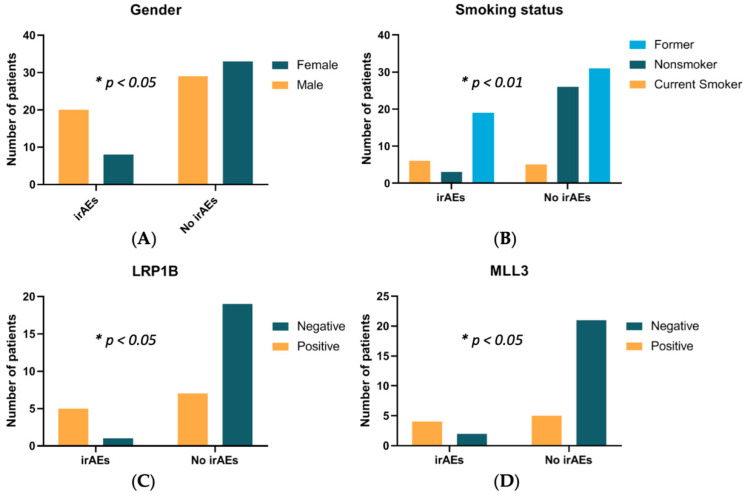
Significant clinical and molecular features with irAEs. (**A**) Association of irAEs with gender, (**B**) smoking status, (**C**) genomic alterations in *LRP1B*, and (**D**) *MLL3*. * Fisher’s exact test by GraphPad.

The irAE group comprised more male patients (*n* = 20, 71.4% vs. *n* = 29, 46.8%; *p* < 0.05; Figure 2A), and more current smokers (*n* = 6, 21.4% vs. *n* = 5, 8.1%; *p* < 0.01; Figure 2B) and former smokers (*n* = 19, 67.9% vs. *n* = 31, 50%; *p* < 0.01; Figure 2B). The non-irAE group comprised more never smokers (*n* = 26, 41.9% vs. *n* = 3, 4.8%; *p* < 0.01; Figure 2B). Seventeen (60.7%) patients in the irAE group underwent invasive diagnostic procedures during hospitalization, including bronchoscopy (*n* = 6, 21.4%), esophageal gastroscopy/colonoscopy (*n* = 5, 17.9%), thoracentesis (*n* = 2, 7.1%), liver biopsy (*n* = 1, 3.6%), skin biopsy (*n* = 1, 3.6%), kidney biopsy (*n* = 1, 3.6%), and brain surgery (*n* = 1, 3.6%) as shown in Table 3. In the non-irAE group, 25 (40.3%) patients underwent thoracentesis (*n* = 8, 12.9%), bronchoscopy (*n* = 6, 9.7%), EGD/colonoscopy (*n* = 6, 9.7%), liver biopsy (*n* = 3, 4.8%), brain surgery (*n* = 2, 3.2%), spine surgery (*n* = 1, 1.6%), and pericardium biopsy (*n* = 1, 1.6%). 

**Figure 3:**
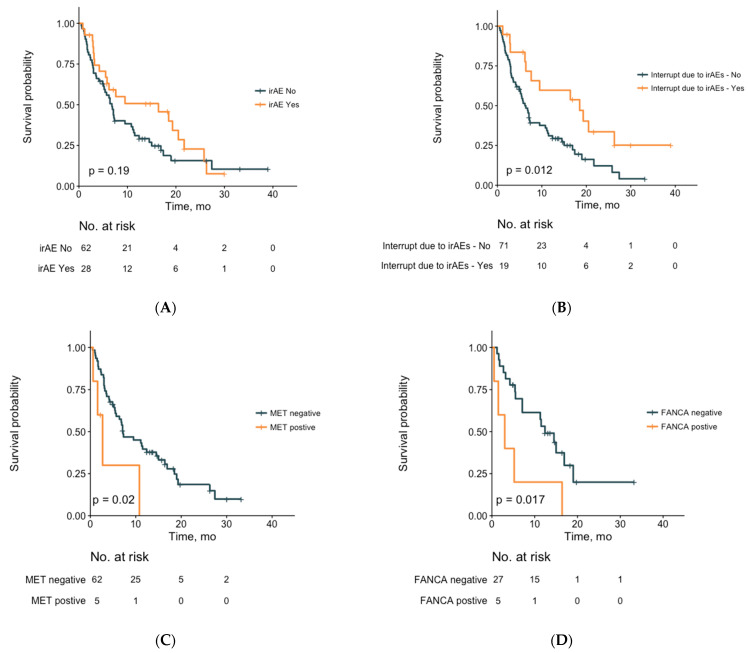
Clinical and molecular features with OS. (**A**) OS with irAEs, (**B**) interruption of ICIs due to irAEs, (**C**) genomic alterations in *MET*, and (**D**) *FANCA*. Log-rank (Mantel–Cox) tests were used to compare the survival curves. Data visualization and statistical analysis were performed with R.

IrAE and non-irAE development were not associated with statistically significant superior OS (Figure 3A). However, we observed a trend toward significance in OS with patients who experienced irAEs compared to those who did not experience irAEs (median 16.4 vs. 6.8 months, *p* = 0.19, Figure 3A). A significant OS benefit was confirmed by multivariate analysis for irAE patients (*n* = 19/28, 67.9%) who underwent ICI treatment interruption due to irAE occurrence (*n* = 19/90, 21.1% vs. *n* = 71/90, 78.9%; median 18.5 vs. 6.2 months; *p* < 0.05) and visualized on survival curves (HR with 95% CI: 0.47 (0.28–0.79); *p* < 0.05; Figure 3B). Patients on the first line of ICI therapy had significantly longer OS than those on second-line or greater ICI therapy (*p* < 0.01).

### 3.3. Molecular Features in irAE and Non-irAE Population

In the overall population, *TP53* ranked as the most detected GA (*n* = 40/66, 60.6%) followed by *LRP1B* (*n* = 12/32, 37.5%), *KRAS* (*n* = 23/77, 29.9%), *MLL3* (*n* = 9/32, 28.1%), *EGFR* (*n* = 16/77, 20.8%), and *PIK3CA* (*n* = 9/66, 13.6%). We analyzed the association between recurrent tumor mutations and irAE occurrence (Table 4). We observed the enrichment of *LRP1B* mutations (*n* = 5/6, 83.3% vs. *n* = 7/26, 26.9%; OR (95% CI) = 13.5 (1.7–166.1), *p* < 0.05; Figure 2C) and MLL3 mutations (*n* = 4/6, 66.7% vs. *n* = 5/26, 19.2%; OR (95% CI) = 8.4 (1.3–49.3), *p* < 0.05; Figure 2D) in irAE patients compared to non-irAE patients. 

However, no statistically significant difference was found in the *MLL3* or *LRP1B* mutation status corresponding with irAE occurrence in our multivariate logistic regression analysis (Table 5). The most frequent GAs and patient demographic information are visualized in the oncoplot in Figure 1. Patients with *MET* (*n* = 5/67, 7.5%) or *FANCA* GAs (*n* = 5/32, 15.6%) demonstrated shorter median OS compared to patients without *MET* (median 2.7 vs. 7.2 months; HR with 95% CI: 3.1 (0.57–17.1), *p* < 0.05) or *FANCA* GAs (median 3.0 vs. 12.4 months; HR with 95% CI: 3.1 (0.70–13.8); *p* < 0.05) (Figure 3D). This relationship between OS and GAs in *MET* (HR, 3.06; 95% CI, 1.08–8.65; *p* < 0.05) and *FANCA* (HR, 3.31; 95% CI, 1.22–9.04; *p* < 0.05) was retained in the multivariate Cox analysis (Table 6). 

## 4. Discussion

The use of ICIs in lung cancer treatment has drastically improved the outcomes of advanced NSCLC patients with an average five-year OS of 15.6% with nivolumab and 23.2% with pembrolizumab as a first-line therapy [28,29]. However, patients who undergo ICI treatment can experience hospital admissions due to severe irAEs and/or other comorbidities. As researchers continue to investigate ICI treatment in earlier-stage disease, it is necessary to explore strategies in minimizing toxicities and avoiding severe irAEs that could be long-lasting or fatal. In this study, we analyzed 90 patients with thoracic cancers who were hospitalized during ICI treatment. Of those, 28 patients (31.1%) experienced irAEs with the most common irAE being pneumonitis (*n* = 10/90, 11.1%). 

This is consistent with other reports demonstrating that 12% of emergency room visits and inpatient care were associated with irAE development in metastatic solid tumor patients undergoing ICI treatment [30]. This result is also consistent with a previous study that reported immune-related interstitial pneumonia as the most common irAE in 13.2% (*n* = 5/38) of lung cancer patients treated with nivolumab [31]. 

We also reported that patients with documented irAEs underwent more invasive diagnostic procedures but with no observed difference in the hospital LOS. The severity of irAEs may have caused further intensive interventions due to the risk of long-lasting effects. Sattar et al. described a correlative study between irAEs and efficacy in an older patient population treated with ICIs, and patients age ≥75 years did not present with excess toxicities [32], consistent with our findings of no associations between irAE development and age. 

However, we did not observe an OS benefit between our irAE and non-irAE populations. Previous studies have demonstrated superior progression-free survival (PFS) and OS in patients with irAEs, while our study only demonstrated a trend toward significance for OS in our irAE group [31,33,34,35]. In a large observational study, Grangeon et al. measured the survival outcomes in 270 patients with metastatic NSCLC treated with at least one dose of anti-PD-L1 or anti-PD-1 antibodies. The study stratified cohorts between patients who did and did not experience irAEs. 

Correspondingly, superior PFS and OS were seen in the cohort who experienced irAEs compared to those who did not experience irAEs (OS: not reached (NR) versus (vs) 8.21 months (hazard ratio (HR) 0.29; 95% confidence interval (CI) 0.18–0.46; *p* = 0.001); PFS: 5.2 vs. 1.97 months (HR 0.42; 95% CI 0.32–0.57; *p* < 0.001)). Interestingly, other measures such as the overall response rate (ORR) (22.9% vs. 5.7%, *p* < 0.0001) and disease control rate (DCR) (76% vs. 58%, *p* < 0.001) were also lengthened in the irAE-positive vs. non-irAE cohorts [36]. In our cohort, we did not observe any survival benefit with the use of corticosteroids. 

Interestingly, Haratani et al. showed that patients who required systemic corticosteroids for irAE management had superior survival outcomes, while Shafqat et al. [35]. demonstrated that irAEs were associated with improved PFS regardless of systemic corticosteroids use [35,37]. The 19 patients who had discontinuation or interruption of ICIs due to irAEs had significantly longer OS, which implied the positive correlations of irAEs with survival outcomes. However, in clinical practice, people might be more comfortable to stop treatment when their disease is better-controlled; therefore, this might be a highly selective patient subpopulation. 

Our results demonstrated an OS benefit for patients who underwent ICI treatment as first-line compared to second-line or greater (*p* < 0.01). A study by Durbin et al. confirmed our results by showing a shorter OS in metastatic solid tumor patients who underwent ICI treatment as second-line or greater [38]. However, another study also analyzed the safety and efficacy of ICIs as second-line treatment in a real-world setting. Chen et al. described the association between the occurrence of irAEs and higher PFS in a patient population who received ICIs in the second-line setting and concluded that the presence of irAEs may act as a predictive marker for antitumor efficacy [39]. 

Next, our study revealed that patients who experienced an interruption of ICI treatment due to irAEs had significantly longer OS than those who continued treatment (*p* < 0.05), suggesting a positive correlation between irAE occurrence and survival outcomes. Conversely, Ksienski et al. showed that treatment interruptions in NSCLC patients undergoing treatment with pembrolizumab or nivolumab due to documented irAEs (*n* = 116/271, 42.8%) were associated with a worse OS [40]. 

A correlative study by Mouri et al. retrospectively analyzed 49 NSCLC patients treated with nivolumab that had treatment interruption due to a serious irAE. With patients stratified into a retreatment or discontinuation cohort, patients rechallenged with nivolumab displayed an ORR of 15%, without a significant increase in irAEs; however, the median OS and PFS did not differ significantly among the patient cohorts [41].

The difference among survival outcomes with varying ICIs used for treatment may also play a role in discontinuation if the patient experiences detrimental irAEs. Lastly, Jia et al. describe varying biomarkers that can predict irAEs based on specific and nonspecific symptoms. Due to irAE effects in every organ, ongoing investigation in regards to the application scope, benefit from treatment interruption, and selection of the treatment population for ICIs based on biomarkers is required [42].

Our analysis reported enrichment of somatic *LRP1B* and *MLL3* mutations in patients with irAEs. Yet, it was not statistically significant in the multivariate analysis, likely due to the limited sample size and confounding factors of smoking and gender. *LRP1B* gene encodes for an LDL receptor and acts as a putative tumor suppressor in lung cancer whose function is only partially defined [43,44]. Chen et al. reported greater survival and higher TMB in melanoma and NSCLC patients with *LRP1B* mutations undergoing ICI treatment [45]. *MLL3* gene encodes for histone 3 lysine 4 methyltransferases and acts as a tumor suppressor. 

Mutated MLL3 (or KMT2C) proteins have been implicated in multiple cancers, including urothelial carcinoma, human lymphoid, and myeloid leukemia [46,47,48]. Further, we found that NSCLC patients with *FANCA* mutations had significantly worse OS compared to those without *FANCA* mutations. The *FANCA* gene is important for the repair of double-stranded DNA breaks and is involved in the cellular process known as the Fanconi anemia pathway. Outcomes have been assessed in patients treated with ICIs, and the results showed significantly higher objective response rate, longer median PFS, and longer median OS with patients on PD-(L)1 therapy [49]. 

The survival outcome that we observed was also previously confirmed in a larger cohort by our group, yet further investigation is required [50]. The *MET* gene encodes a transmembrane receptor tyrosine kinase, and its ligand hepatocyte growth factor (HGF) is involved in the MET/HGF signaling pathway. Patients in our cohort with *MET* GAs were associated with poor OS, and the statistical significance was retained in the multivariate analysis. 

This is consistent with the previous findings of *MET* mutated lung cancer and worsened outcomes with immunotherapy treatment [24,51]. We did not observe a correlation between TMB with irAEs or OS, which may be explained by the lack of TMB information in our cohort as only 18 patients had TMB information available. It is unclear how somatic mutations in tumors contribute to the development of irAEs, and more research is warranted to examine the role of genomic mutations in lung cancer immunotherapy.

## 5. Conclusions

Our retrospective analysis investigated the clinical and molecular features of lung cancer patients undergoing ICI treatment who were hospitalized. We observed that patients with irAE occurrences who required treatment interruption had a significantly longer OS. Further, patients with somatic GAs in *FANCA* and *MET* had a worse OS, which is consistent with previously reported studies. A limitation of this study is that the patient cohort was of limited sample size and from a single institution. Further investigation is required to analyze a larger and diverse population set. Strikingly, our findings indicated that the majority of patients who were hospitalized on ICI treatment did not have an irAE. Therefore, future clinical studies should focus on identifying and cataloging the variables that may be associated with hospitalization due to ICI treatment. 

## Figures and Tables

**Table 1:** List of irAEs.

IrAEs	No (%)
Pneumonitis	10 (11.1%)
Adrenal insufficiency	4 (4.4%)
Hypothyroidism	4 (4.4%)
Colitis	4 (4.4%)
Liver injury	3 (3.3%)
Nephritis	2 (2.2%)
Heart failure	1 (1.1%)
Pancreatitis	1 (1.1%)
Diabetic ketone acidosis	1 (1.1%)
Arthralgia	1 (1.1%)
Rash	2 (2.2%)
Other/Infection	2 (2.2%)
Multiple irAEs	7 (7.8%)
Total patients with irAEs	28 (31.1%) ^1^

^1^ 90 patients had hospital admissions during ICI treatment.

**Table 2:** Baseline patient characteristics.

Characteristics	*n* = 90 (%)	IrAEs *n* = 28 (%)	No irAEs *n* = 62 (%)	*p* Values ^1^
Median age at ICI (range 36–88)	68.5	70.5	67.5	ns
Gender				<0.05
Women	41 (45.6%)	8 (28.6%)	33 (53.2%)	
Men	49 (54.4%)	20 (71.4%)	29 (46.8%)	
Smoking status				<0.01
Current	11 (12.2%)	6 (21.4%)	5 (8.1%)	
Former	50 (55.6%)	19 (67.9%)	31 (50.0%)	
Never	29 (32.2%)	3 (10.7%)	26 (41.9%)	
Histology				<0.01
Lung adenocarcinoma	63 (70%)	16 (57.1%)	47 (75.8%)	
Lung squamous	14 (15.6%)	4 (14.3%)	10 (16.1%)	
SCLC	5 (5.6%)	5 (17.9%)	0	
Others ^2^	8 (8.9%)	3 (10.7%)	5 (8.1%)	
ICIs with other therapy				ns
Yes	12 (13.3%)	2 (7.1%)	10 (16.1%)	
No	78 (86.7%)	26 (92.9%)	52 (83.9%)	
PD-L1				ns
Negative	16 (17.8%)	5 (17.9%)	11 (17.7%)	
1% to <50%	8 (8.9%)	1 (3.6%)	7 (11.3%)	
50% and above	21 (23.3%)	7 (25.0%)	14 (22.6%)	
Not tested	45 (50.0%)	15 (53.6%)	30 (48.4%)	
Median lines of therapy (range 1–7)	2	2	2	ns
Brain metastasis				ns
Yes	35 (38.9%)	10 (35.7%)	25 (40.3%)	
No	55 (61.1%)	18 (64.3%)	37 (59.7%)	
Median length of stay (range 1–37)	7	7	6	ns

^1^ Chi-square test and Fisher’s exact test. ns, not significant. ^2^ Others: 1 poorly differentiated tumor including NSCLC, not otherwise specified (NSCLC-NOS), 1 large cell lung cancer, 1 lung atypical carcinoid, 1 adenosquamous tumor, 1 mixed large cell with neuroendocrine tumor, 1 small cell transformed lung adenocarcinoma, 1 mixed adenocarcinoma with large cell neuroendocrine tumor, and 1 mesothelioma.

**Table 3:** Invasive procedures after ICIs.

Invasive Procedures after ICIs	irAEs (*n* = 28)	No irAEs (*n* = 62)
Bronchoscopy/lung biopsy	6 (21.4%)	6 (9.7%)
EGD/Colonoscopy	5 (17.9%)	6 (9.7%)
Thoracentesis	2 (7.1%)	8 (12.9%)
Liver biopsy	1 (3.6%)	3 (4.8%)
Skin biopsy	1 (3.6%)	0
Kidney biopsy	1 (3.6%)	0
Brain surgery	1 (3.6%)	2 (3.2%)
Spine surgery	0	1 (1.6%)
Pericardium biopsy	0	1 (1.6%)
Total	17 (60.7%)	25 (40.3%) ^1^

^1^ Chi-square test *p* < 0.05. The total number of patients who had invasive procedures, including one patient who had a lung biopsy, thoracentesis, and pericardium biopsy.

**Table 4:** Mutations and their associations with irAEs.

Genomics	All (%)	IrAEs (%)	No irAEs (%)	OR (95% CI)	*p* Values ^1^
TP53					ns
Positive	40	10 (66.7%)	30 (58.8%)		
Negative	26	5 (33.3%)	21 (41.2%)		
Not tested	24	13	11		
KRAS					ns
Positive	23	8 (40%)	15 (26.3%)		
Negative	54	12 (60%)	42 (73.7%)		
Not tested	13	8	5		
EGFR					ns
Positive	16	2 (10%)	14 (24.6%)		
Negative	61	18 (90%)	43 (75.4%)		
Not tested	13	8	5		
LRP1B				13.5 (1.7–166.1)	<0.05
Positive	12	5 (83.3%)	7 (26.9%)		
Negative	20	1 (16.7%)	19 (73.1%)		
Not tested	58	22	36		
PIK3CA					ns
Positive	9	3 (20%)	6 (11.8%)		
Negative	57	12 (80%)	45 (88.2%)		
Not tested	24	13	11		
MLL3				8.4 (1.3–49.3)	<0.05
Positive	9	4 (66.7%)	5 (19.2%)		
Negative	23	2 (33.3%)	21 (80.8%)		
Not tested	58	22	36		
TMB					ns
TMB-Low	5	1 (33.3%)	4 (26.7%)		
TMB-Intermediate	9	1 (33.3%)	8 (53.3%)		
TMB-High	4	1 (33.3%)	3 (20%)		
Not tested	72	25	47		

**Table 5:** Risk factors for irAEs by multivariate analysis.

Risk Factors	Odds Ratio (95% CI)	*p* Values ^1^
Gender		
Female	References	
Male	1.47 (0.43–4.99)	0.5358
Smoking		
Never	References	
Current	3.61 (0.43–30.11)	0.2363
Former	3.44 (0.76–15.45)	0.1073
MLL3		
Negative	References	
Positive	6.52 (0.70–60.62)	0.0991
LRP1B		
Negative	References	
Positive	8.00 (0.65–98.01)	0.1037

^1^ Multivariate logistic regression for irAEs by R (excluding small cell lung cancer).

**Table 6:** Multivariate analysis for OS (*n* = 90).

Risk Factors	HR (95%CI)	*p* Values ^1^
Gender		
Female	Reference	
Male	1.11 (0.57–2.15)	ns
IrAEs		
No	Reference	
Yes	1.21 (0.50–2.92)	ns
Interrupt ICIs due to irAEs		
No	Reference	
Yes	0.05 (0.01–0.19)	<0.001
Lines of therapy		
≥3 lines	Reference	
2nd line	0.62 (0.25–1.49)	
1st line	0.21 (0.07–0.58)	<0.01
EGFR		
Negative	Reference	
Positive	1.45 (0.55–3.77)	ns
FANCA		
Negative	Reference	
Positive	11.30 (3.36–38.01)	<0.001
MET		
Negative	Reference	
Positive	11.17 (2.92–42.81)	<0.001

^1^ Multivariate Cox proportional hazards model for OS (excluding small cell lung cancer).

## Data Availability

The data presented in this study are available on request from the corresponding author.

## Abbreviations

CI	Confidence interval
CTLA-4	Cytotoxic T-lymphocyte–associated antigen 4
DCR	Disease control rate
EGD	Esophagogastroduodenoscopy
FANCA	Fanconi anemia complementation group A
FDA	Food and Drug Administration
GAs	Genomic alterations
HGF	Hepatocyte growth factor
HR	Hazard ratio
ICIs	Immune checkpoint inhibitors
IHC	Immunohistochemistry
IrAEs	Immune-related adverse events
IRB	Institutional review board
KMT2C	Lysine (K) methyltransferase 2C
LRP1B	Low-density lipoprotein (LDL) receptor-related protein 1B
LOS	Length of stay
MLL3	Mixed-lineage leukemia protein 3
NGS	Next-generation sequencing
NSCC-NOS	Non-small cell carcinoma, not otherwise specified
NSCLC	Non-small cell lung cancer
OR	Odds ratio
ORR	Overall response rate
OS	Overall survival
PD-1	Program death -1
PD-L1	Programmed death-ligand 1
PFS	Progression-free survival
SCLC	Small cell lung cancer
TMB	Tumor mutation burden
VS	Versus
